# Non-arteritic ischemic optic neuropathy followed by intravitreal bevacizumab injection: Is there an association?

**DOI:** 10.4103/0301-4738.64142

**Published:** 2010

**Authors:** Ali A Bodla, Prasad Rao

**Affiliations:** Eye Unit, Royal Shrewsbury Hospital, Mytton Oak Road, Shrewsbury, SY3 8XQ, UK

**Figure 1:**
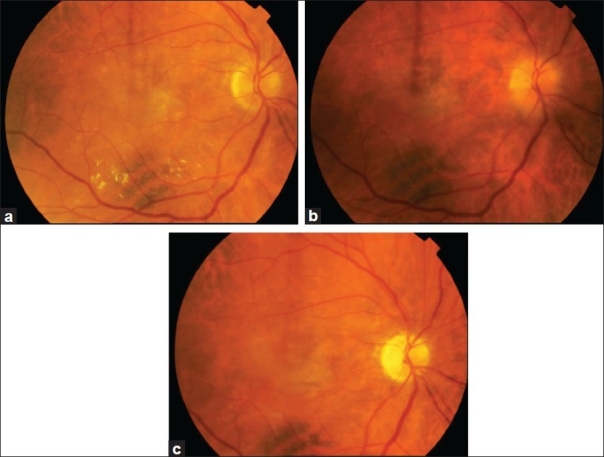
Serial photograph of the right eye showing normal optic disc (a) before the initiation of treatment, optic disc swelling (b) a month after the third injection and evident optic atrophy five months (c) after the onset of NAION

Dear Editor,

A 70-year-old lady was seen in the retina clinic following a referral for suspected bilateral occult choroidal neovascular membranes (CNV). Her symptoms included intermittent blurred vision. Best corrected visual acuity was 20/60 in the right and 20/30 in her left eye. Fundus examination revealed a central area of cystoid macular edema surrounded by exudates and hemorrhages in her right eye and an area of serous pigment epithelial detatchment in the left eye. She had a healthy optic nerve with distinct disc margins [Fig. 1a]. Fluorescein angiography showed an active subfoveal neovascular lesion in the right eye. No such lesion was identified in her left eye but there was a combination of serous fluid and atrophic changes confirmed as pigment epithelial detatchment. Right eye optical coherence tomography (OCT) examination revealed thickened and elevated retinal layers at the macula due to subretinal fluid and a subfoveal hyper-reflective mass consistent with an active membrane. The patient was treated with three uneventful intravitreal injections of 1.25 mg bevacizumab, each a month apart. Despite early regression in the size of CNV after the first intravitreal injection macular edema and leakage from CNV persisted. Complete CNV regression subsequently resulted after the third and last injection, with resulting improvement in vision to 20/30. She subsequently presented with sudden onset blurred vision in her right eye, a month following the last injection of intravitreal bevacizumab. Her visual acuity had dropped down to 20/120 in the same eye along with a relative afferent papillary defect. Fundus assessment was consistent with optic disc swelling of a pallid nature and nearby cotton-wool spots [Fig. 1b]. Visual field assessment revealed a classical superior altitudinal field defect. Findings in her case were classical for a case of non-arteritic ischemic optic neuropathy (NAION) [Figs. 1b and c]. She had no constitutional symptom or sign suggestive of an arteritic component. Her erythrocyte sedimentation rate, C reactive protein and platelet count were found to be in normal range. Fundus flourescien angiography revealed a normal choroidal filling time ruling out any lesion of vasculitic nature.

There were no identifiable risk factors for NAION in her case apart from the age. Intraocular pressures were found to be normal during pre and post-treatment phase. One possible explanation can be a compromise in the vascular integrity of the optic nerve vessels as vascular endothelial growth factor (VEGF) participates in the maintenance of vascular systems in adults.[1] To date, pegaptanib and ranibizumab in humans have not shown adverse effects on normal retinal or choroidal vasculature but bevacizumab has been proven to be more potent than its counterparts in the terms of a longer half life and much higher systemic levels.[2] We also want to highlight the direct neuroprotective role of VEGF which again is compromised with the use of bevacizumab.[3] Blockage of VEGF with bevacizumab has been associated with stroke and reversible focal posterior leukoencephalopathy of the brain.[4] It is known to cause mitochondrial damage in the inner segments of photoreceptors and apoptosis in the retina.[5] In animal models VEGF inhibition has been implicated in diabetic and ischemic neuropathy.[2] Chronic inhibition of VEGF-A in normal adult animals resulted in loss of retinal ganglia.[2,5] We are aware of the fact that this could have been a coincidence but still strongly believe that it is not possible to ignore the compromised vascular integrity and lack of neuroprotection as a possible cause of NAION in our patient. We need to take into consideration the frequency of drug administration especially in the case of bevacizumab due to its presumed higher potency.
